# What learning strategies influence higher-order learning behaviours of medical students?

**DOI:** 10.1080/07853890.2023.2205166

**Published:** 2023-05-12

**Authors:** Jun Liu, Kaiwen Yuan, Xun Lin, Wenjia Zhu

**Affiliations:** a Institute of Governance Modernization, Shanghai University of Engineering Science, Shanghai, China; b Foreign Language Teaching Center, Shanghai University of Traditional Chinese Medicine, Shanghai, China; c Shuguang Hospital Affiliated to Shanghai University of Traditional Chinese Medicine, Shanghai, China; d Innovation and Entrepreneurship Education College, East China Normal University, Shanghai, China

**Keywords:** Medicine, learning behaviour, learning strategy, higher-order learning, Chinese College Student Survey (CCSS)

## Abstract

**Aims:**

The aim of this study was to explore the relationship between the application of learning strategies and the emergence of higher-order learning behaviours among medical students in Chinese provincial undergraduate colleges, while also examining the impact of social demographic variables on the development of higher-order learning behaviours and learning strategy preferences.

**Methodology:**

We conducted a relevant cross-sectional study using the Chinese College Student Survey (CCSS) online questionnaire to evaluate higher-order learning behaviours and learning strategies in medical undergraduate students attending provincial colleges in China. A total of 992 valid questionnaires were collected and analysed using SPSS 22.0 (SPSS Inc., Chicago, IL). We performed statistical analysis using one-sample *t*-tests to compare the results with the national norm score for medical subjects in undergraduate colleges. We also conducted variance analysis and regression analysis.

**Results:**

The study found that the average scores for higher-order learning behaviours, enquiry-based learning and receptive learning behaviour among medical undergraduate students in provincial colleges were higher than the national norm score for medical subjects, indicating a positive trend. However, the average scores for other indicators were lower than the national norm score. The utilization of learning strategies and the development of higher-order learning behaviours among students were affected by various factors such as grade and gender. The study suggests that the preference for certain learning strategies, such as enquiry-based, receptive, integrative and collaborative, can have a significant impact on the emergence of higher-order learning behaviours.

**Conclusions:**

The study has demonstrated a positive correlation between the utilization of learning strategies and the development of higher-order learning behaviours. This relationship has been observed in medical students attending provincial undergraduate colleges, where the adoption of enquiry-based, receptive, integrative and collaborative learning strategies has been found to significantly influence the emergence of higher-order learning behaviours.

**KEY MESSAGES**
- The implementation of learning strategies among medical students in provincial undergraduate colleges in China has a significant impact on high-level learning behaviours.
- The impact of high-level learning behaviours is reliant on comprehensive support from four distinct learning strategies: receptive learning, inquiry-based learning, comprehensive learning and collaborative learning.
- One of the most impactful learning strategies is receptive learning, particularly on high-order learning behaviours. On the other hand, reflective learning does not seem to have a significant effect.
- Changes in grades can significantly impact higher-order learning behaviours and affect the propensity for reflective and collaborative learning strategies.
- Females generally exhibit a greater preference for receptive learning strategies, while males tend to exhibit a greater preference for inquiry-based learning strategies

## 1. Introduction

The process of student learning involves not only physiological and psychological functions but also cultural traditions and mental habits. The colleges in Europe and the United States have identified certain high-order learning characteristics through long-term teaching practices, such as internal learning interest, deep knowledge understanding, active classroom questioning and questioning of teachers’ views. Based on these behavioural characteristics, experts have developed standards to evaluate learning behaviour, including active learning, deep learning and self-regulated learning. Higher-order learning strategies go beyond simply learning knowledge and skills; they also emphasize purposeful and adaptive application of learning strategies in response to environmental changes. In contrast, lower-order learning focuses on short-term memory enhancement through mechanical means. Higher-order learning involves complex cognitive processes and places greater emphasis on the four higher-order thinking abilities: analysis, integration, evaluation and application. Bloom’s Taxonomy of Learning Domains provides the mainstream definition of higher-order learning, dividing learning into six levels: creating, understanding, applying, analysing, remembering and evaluating [1–3]. Anderson further developed this theory, replacing nouns with verbs to describe cognitive processes [4]. The six levels of cognitive behaviour proposed by Anderson, from lower to higher-order, are memorization, understanding, application, analysis, evaluation and creation [1,5].

According to the National Survey of Student Engagement (NSSE), advanced learning requires a shift from simple memorization and understanding to deep learning involving synthesis, application and analysis. This change encourages learners to step out of their comfort zone, develop higher-order thinking modes and enrich their learning experience. To adapt to this shift, students develop behavioural strategies that are tailored to the challenges of the task, involving the selection of methods, techniques and study plans. Students’ learning experience and behaviour have a more significant impact than the content of lecturers’ classroom teaching [6].

In China, scholarly articles on learning behaviours and strategies tend to primarily focus on introducing foreign scientific theories and teaching methods for primary and secondary education. While research on the application of learning behaviours and strategies for college students is more common in distance education scenarios or broader topics, there is a noticeable lack of empirical studies in the classroom context [7]. Furthermore, there is currently no empirical research on the learning behaviours and strategies of college students in medical education.

In medical education, the core competency of students is to develop comprehensive and sustainable learning capabilities. High-quality learning is essential for producing high-quality and sustainable learning outcomes, which are necessary for guaranteeing the quality of medical education. Higher-order learning behaviours are an example of high-quality learning input behaviour, which can diagnose the learning quality of medical students in provincial undergraduate colleges from multiple dimensions [8]. Given that provincial colleges in China bear the primary responsibility of training medical talents and that medical students from these colleges will comprise the main group of future doctors, the empirical evidence resulting from the assessment of higher-order learning behaviours can serve as a basis for provincial undergraduate colleges to implement more targeted teaching optimization and stimulate the learning potential of medical students [9].

In this research, the Chinese College Student Survey (CCSS) 2020 was employed as a survey instrument to address the following research inquiries: What are the present features of higher-order learning behaviour demonstrated by medical undergraduates in Chinese colleges? To what extent are students capable of applying their acquired learning strategies? Is there a significant correlation between these two aspects? This investigation concentrates on an essential cohort of medical undergraduates in provincial colleges across China and exposes the current state and challenges faced by Chinese medical students, with a focus on their learning behaviour and strategies. To the best of our knowledge, this is the first study to explore the relationship between learning strategies and higher-order learning behaviours among medical undergraduates in China.

## 2. Materials and methods

The present study utilized the CCSS 2020 questionnaire to administer a cross-sectional survey among clinical medicine undergraduates in various provincial undergraduate colleges in China, including Shanghai, Jiangsu and Guangdong. The principal objective of this research was to assess the association between higher-order learning behaviours and the implementation of distinct learning strategies among medical students. The study was conducted in compliance with the ethical standards set forth in the World Medical Association Declaration of Helsinki and was granted an exemption by the Ethics Committee of Shanghai University of Traditional Chinese Medicine (211201).

The study’s sample was selected using the grade-stratified random sampling technique. A total of 1100 questionnaires were dispensed among clinical medical undergraduates, of which 992 were deemed suitable for analysis. This study employs the online survey platform developed by Tsinghua University. The platform is customized according to the features of the Chinese university-college dual management system that governs the two-level student management. This customization expedites data collection procedures and guarantees accuracy and scientific soundness. The questionnaire was disseminated to participants anonymously via an online survey platform (http://ccss.ioe.tsinghua.edu.cn/user/login?), and prior to the formal commencement of the questionnaire, all respondents granted informed consent by clicking the ‘confirm’ button.

### 2.1. Conceptual or theoretical framework

In ‘Cambridge Learning Science Manual,’ Sawyer (2006) emphasizes that deep learning does not reject low-order learning strategies but presents thinking in a gradient of learning activities, which includes both high-order and low-order learning strategies [4]. By employing high-order learning strategies, students can significantly enhance their learning outcomes. Higher-order learning is exemplified by learners’ comprehensive and profound processing of learning materials, encompassing higher-order thinking processes such as comprehension, connection and integration. Such processes cover learning behaviours including analysis, synthesis, judgment and application. Wiggins, in ‘UNDERSTANDING BY Design,’ proposes that low-order learning serves as the foundation on which high-order learning is cultivated. Therefore, the crucial aspect of learning activities is to achieve advanced thinking. The ultimate goal of learning is to achieve knowledge migration, and the further the distance of the migration, the deeper the students’ understanding of the knowledge.

Consequently, learning behaviour is closely associated with the learning strategy chosen by students. Drawing from the theoretical framework outlined above, this study posits the following research assumptions: there exists a causal relationship between high-order learning behaviour ability and learning strategy application, and a research framework is proposed to investigate the impact of behaviour on learning strategy application.

### 2.2. Measurement tool

The CCSS questionnaire originated from the Chinese version of the NSSE introduced by Tsinghua University. After years of continuous revision, it gradually adapted to the cultural environment of Chinese higher education, and finally formed the CCSS questionnaire.

This study used the CCSS questionnaire to conduct a self-evaluation survey and analysed the parts of higher-order learning behaviours and application learning strategies in the students’ learning diagnosis module. The part of higher-order learning behaviour mainly investigates the situation of students’ analysis, synthesis, judgment and application of learning behaviour. The part of learning strategy application mainly investigates students’ learning and cognitive behaviours in and out of class.

According to the CCSS questionnaire provided by Tsinghua University, the reliability of each indicator of the student learning diagnosis module is as follows:

Learning strategy application indicators: (1) receptive learning Cronbach’s *α* = 0.78; (2) enquiry-based learning Cronbach’s *α* = 0.816; (3) reflective learning Cronbach’s *α* = 0.841; (4) integrative learning Cronbach’s *α* = 0.758; (5) collaborative learning Cronbach’s *α* = 0.777.

Higher-order learning behaviour indicators: Cronbach’s *α* = 0.813.

Student self-harvest report indicator: Cronbach’s *α* = 0.936.

At the same time, in the process of developing the CCSS questionnaire, students’ self-reported harvest indicator was used as the criterion to test the criterion-related validity of each index in the learning diagnosis module. The report is as follows:

*Criterion related validity of learning diagnosis module*

**Table:** 

	Higher-order learning	Receptive learning strategies	Enquiry-based learning strategies	Reflective learning strategies	Integrative learning strategies	Collaborative learning strategies
Student self-harvest report	0.454	0.442	0.493	0.541	0.554	0.460

The survey included the following aspects: (1) demographic characteristics, (2) learning strategies and (3) higher-order learning behaviours.

Demographic characteristics include gender (male, female), grade (freshman, sophomore, junior, senior, fifth, sixth and seventh).

Learning strategies consists of five secondary indicators: receptive learning, enquiry-based learning, reflective learning, integrative learning and collaborative learning. It include receptive learning (four questions), enquiry-based learning (six questions), reflective learning (five questions), integrative learning (four questions) and collaborative learning (four questions). Learning strategies were evaluated from four scales: (1) never, (2) sometimes, (3) often, (4) very often, with high or low scores indicating the frequency of occurrence, and the higher the score, the higher the occurrence frequency.

There are four questions in higher-order learning behaviours. We used four grades to evaluate every question: (1) not emphasized, (2) partly, (3) normal, (4) highly, the score indicates the degree of agreement with the expression, the higher the score, the closer it is to the actual situation.

Information on the specificity of the questionnaire is provided in the supplementary appendix.

### 2.3. Data collection: source or procedure

WeChat, a popular mobile app in China, has gained widespread adoption due to its multifaceted features, including real-time chat, group communication, application services and payment processing capabilities. It is similar to WhatsApp.

In order to conduct research and investigation among college students, we established the groups of WeChat based on students’ grades. The methodology employed in conducting the survey entailed the determination of a sampling list based on grades, followed by the formation of WeChat groups per grade level. The purpose of these groups was to notify students to participate in the online survey. The groups of WeChat are expected to serve as a vital platform for students to collaborate and communicate on research and investigation. To join the groups of WeChat, students can either scan the group’s QR code or request an invitation to join the groups. An invitation letter was initially sent out via the WeChat groups, and after a three-day period, the survey link was distributed to students via WeChat and email.

The links were resent twice over a period of two weeks. The survey was concluded one week after the final distribution of the links, and data collection was terminated at that point. In order to ensure the anonymity of participants, each student was granted access to only one online survey, and was required to confirm their informed consent prior to participation. To aid students in understanding the details of the study and to provide assistance in completing the questionnaire, the researchers established a consultation WeChat account.

### 2.4. Data analysis

The descriptive analysis was conducted to determine the mean and standard deviation of the continuous variables of higher-order learning behaviours and the five learning policy indicators. The normality of these variables was tested using the *K*–*s* test. The results of the test indicated that the *Z*-score of the variables was in the range of 2, which indicated that they were approximately normally distributed.

In this study, descriptive analysis was employed to calculate the mean ± standard deviation of the continuous variables related to higher-order learning behaviours and secondary indicators, such as receptive learning strategy, enquiry-based learning strategy, integrative learning strategy and collaborative learning strategy. The normality of the distributions of higher-order learning behaviours and the scores on the five learning policy indicators was tested through the use of the *K*–*s* test. The normality test showed that the *Z*-score of the continuous variables was within the range of 2, which was deemed to be consistent with the approximate normal distribution standard.

To compare the differences between the means of the above-mentioned continuous variables and the norm standard of Chinese students, a single-sample *t*-test was used. Analysis of variance (ANOVA) was conducted to evaluate the impact of grade and gender on the continuous variables. Pearson’s correlation was employed to assess the correlation between higher-order learning behaviours and continuous variables with approximately normal distributions, such as receptive learning strategies, enquiry-based learning strategies, reflective learning strategies, integrative learning strategies and collaborative learning strategies. The effects of receptive learning strategies, enquiry-based learning strategies, reflective learning strategies, integrative learning strategies and collaborative learning strategies on higher-order learning behaviours were analysed through multiple linear regression. The statistical analysis was conducted using the Statistical Package for Social Sciences (SPSS version 22.0, SPSS Inc., Chicago, IL), and the level of statistical significance was set at .05.

### 2.5. Research variables

This study aimed to provide evidence for the improvement of medical education in China, by exploring the relationship between learning strategies and higher-order learning behaviours. By investigating the preferences of different learning strategies, this study aimed to provide insights for the medical education institutions to enhance the higher-order learning ability of students. The findings of this study can serve as a reference for further research in the field of medical education and the development of educational programs.

In this study, the dependent variable, higher-order learning behaviour, was operationalized to encompass several key aspects of students’ learning, including their ability to comprehend basic knowledge elements, synthesize information to form new explanations, evaluate the reliability and value of information, and solve practical problems in new situations. The demographic variable, which served as control variables, consisted of factors such as gender, grade. On the other hand, the independent variable, application of learning strategies, was comprised of five main components: receptive learning, enquiry-based learning, collaborative learning, reflective learning and integrative learning. These variables were considered to be crucial in this study as they reflect both students’ subjective learning preferences and the objective influence of their external learning environment. By examining the relationship between higher-order learning behaviour and the application of learning strategies, this study aimed to shed light on how different learning strategies impact higher-order learning behaviour among medical students in provincial undergraduate colleges in China. The Description of research variables is summarized in Table 1.

**Table 1.** Description of research variables.

Categorical variable		Identification of variable study design	Characteristics of variables
Dependent variables		Higher-order learning behaviour (HOC_HOL)	Time-variant
Predictor variable	Demographics	Gender and grade	Time-invariant
	Students learning strategy	Receptive learning (LS_TL), enquiry-based learning (LS_EL), reflective learning (LS_RL), integrative learning (LS_IL), cooperative learning (LS_CL)	Time-variant

## 3. Results

The demographic characteristics of the analysis samples (*n* = 992) were as follows: male accounted for 34.9% of the sample (*n* = 346), while female accounted for 65.1% of the sample (*n* = 646); Han nationality students accounted for 75.3% of the sample (*n* = 747), while minority (other) students accounted for 24.7% of the sample (*n* = 245); students with normal length of schooling accounted for 90.3% of the sample (*n* = 896), while students with longer length of schooling accounted for 9.7% (*n* = 96); senior students (4-year and above) accounted for 55% of the sample (*n* = 545), while junior students (1–3 years) accounted for 45% of the sample (*n* = 447).

### 3.1. The status of learning ability

The results of this study indicated that medical students in provincial undergraduate colleges in China exhibited a positive ability for higher-order learning behaviours, with an average score of 55.04, which was slightly higher than the national norm score for medical subjects in undergraduate colleges. This suggests that the higher-order learning abilities of medical students in provincial undergraduate colleges were on par with the national norm level. Moreover, the analysis of the five secondary indicators of learning strategies showed that the scores for the receptive learning strategy and enquiry-based learning strategy were higher than the national norm scores for medical subjects in national undergraduate colleges, which reflects positive performances in these areas. However, the scores for the other three indicators (collaborative learning, reflective learning and integrative learning) were lower than the national norm scores, suggesting negative performances in these areas. These findings are presented in Table 2.

**Table 2.** Overall description of the learning ability of medical students in provincial undergraduate colleges.

Indicator variable	Provincial undergraduate colleges (*X*1)	Minimal	Maximal	Chinese students norms (*X*2)	*X*1 compare *X*2	*p* Value
Receptive learning (LS_TL)	55.44 ± 18.64	8.32	100	52.52 ± 20.88	*X*1 > *X*2	.000***
Enquiry-based learning (LS_EL)	40.79 ± 16.44	5.55	100	36.99 ± 15.31	*X*1 > *X*2	.000***
Reflective learning (LS_RL)	54.32 ± 18.86	13.32	100	56.37 ± 17.25	*X*1 < *X*2	.000***
Integrative learning (LS_IL)	51.34 ± 18.77	8.32	100	51.61 ± 17.82	*X*1 < *X*2	.000***
Collaborative learning (LS_CL)	52.74 ± 17.51	6.66	100	53.04 ± 16.36	*X*1 < *X*2	.000***
Higher-order learning behaviour (HOC_HOL)	55.04 ± 21.66	8.32	100	52.52 ± 20.88	*X*1 > *X*2	.579

The Chinese Norms of medical students from Tsinghua University’s ‘Chinese Undergraduate Study and Development Tracking Survey’.

***p < .001.

### 3.2. The influence of demographic variables

This study examined the influence of demographic variables on higher-order learning behaviours and other learning strategies, including receptive learning, enquiry-based learning, integrative learning and collaborative learning. The independent variables of grade and gender were used to perform statistical tests for differences, as detailed in Tables 3 and 4. The results revealed statistically significant differences between senior and junior students in higher-order learning behaviour, reflective learning strategy and collaborative learning strategy. Furthermore, there were significant differences between males and females in receptive learning strategy and enquiry-based learning strategy.

**Table 3.** The influence of learning strategies with different demographic variables.

		Receptive learning (LS_TL)	Enquiry-based learning (LS_EL)	Reflective learning (LS_RL)	Integrative learning (LS_IL)	Collaborative learning (LS_CL)
Grade	High grade	57.42 ± 18.20	41.99 ± 16.65	57.48 ± 17.96	54.45 ± 18.88	55.22 ± 17.36
	Grade 4	54.17 ± 18.28	42.85 ± 16.39	57.70 ± 17.34	52.48 ± 15.79	56.11 ± 16.54
	Grade 3	55.77 ± 19.05	40.30 ± 15.57	52.46 ± 18.92	50.68 ± 18.62	52.54 ± 17.11
	Grade 1–2	54.02 ± 18.48	39.80 ± 17.44	53.30 ± 19.60	51.61 ± 19.84	49.91 ± 18.14
	*p* Value	0.307	0.354	0.012*	0.220	0.006**
Gender	Male	52.76 ± 18.44	43.19 ± 17.15	56.46 ± 19.18	53.09 ± 18.37	51.95 ± 16.98
	Female	56.42 ± 18.63	39.90 ± 16.10	53.53 ± 18.70	51.51 ± 18.91	52.03 ± 17.71
	*p* Value	0.015*	0.014*	0.056	0.569	0.451

*p < .05 and **p < .01.

**Table 4.** The influence of higher-order learning behaviours with different demographic variables.

	Grade					Gender		
	High grade	Grade 4	Grade 3	Grade 1–2	*p* Value	Male	Female	*p* Value
Higher-order learning behaviour	58.54 ± 21.02	59.23 ± 19.16	53.31 ± 21.23	53.27 ± 23.14	.012*	53.49 ± 21.05	55.61 ± 21.87	.227

* *p *<. 05.

### 3.3. The correlation between higher-order learning behaviour and learning strategies

The study found a positive correlation between the score of higher-order learning behaviour and the indicators of learning strategy application, suggesting that students who were able to apply learning strategies had a higher likelihood of achieving sustainable learning (correlation distance coefficient ranged from 0.263 to 0.499, as shown in Table 5). A regression analysis was also conducted, using the application of learning strategies (receptive learning, enquiry-based learning, reflective learning, integrative learning and collaborative learning) as the independent variable, and higher-order learning behaviours as the dependent variable. The results showed that the reflective learning strategy was not a significant predictor in the regression equation. The remaining learning strategies, sorted from high to low, were enquiry-based learning, receptive learning, integrative learning and collaborative learning. These four learning strategies were found to significantly impact the higher-order learning behaviours of provincial undergraduate medical students (as shown in Table 5).

**Table 5.** Correlation between higher-order learning behaviours with application of learning strategies.

	Higher-order learning behaviour (HOC_HOL)	
	Correlation	*p* Value
Receptive learning (LS_TL)	0.479	.000***
Enquiry-based learning (LS_EL)	0.466	.000***
Reflective learning (LS_RL)	0.423	.000***
Integrative learning (LS_IL)	0.495	.000***
Collaborative learning (LS_CL)	0.499	.000***

***p < .001.

According to the results presented in Table 6, the study found that the application of learning strategies, including receptive learning, enquiry-based learning, integrative learning and collaborative learning, directly impacted the level of higher-order learning behaviour among medical students in provincial colleges.

**Table 6.** Regression analysis of the impact factors of learning strategy for higher-order learning behaviours.

	Estimate	Standard deviation	Wald	df	Significance	Exp (*B*)
Receptive learning (LS_TL)	0.210	0.006	11.034	1	.001**	1.020
Enquiry-based learning (LS_EL)	0.200	0.008	7.070	1	.008**	1.021
Reflective learning (LS_RL)	0.000	0.007	0.001	1	.972	1.000
Integrative learning (LS_IL)	0.027	0.008	11.666	1	.001**	1.027
Collaborative learning (LS_CL)	0.024	0.007	12.269	1	.000***	1.025
Constant	–4.344	0.402	116.548	1	.000	0.013

**p < .01.

***p < .001.

## 4. Discussion

This study aimed to investigate the utilization of learning strategies among medical students in Chinese provincial undergraduate colleges, with a particular focus on the impact of these strategies on higher-order learning behaviours. The study also examined the influence of gender and grade on these learning behaviours and strategies.

### 4.1. The status of high-level learning behaviour

Regarding higher-order learning behaviours, the study found that medical students in provincial undergraduate colleges exhibited a satisfactory level of such behaviours, which exceeded the norm level observed in other Chinese medical students. The students’ performance in postgraduate entrance exams and standardized training over the past few years revealed that students from provincial undergraduate colleges demonstrated strong self-learning capabilities and consciousness, including high self-demands, an earnest learning attitude and positive motivation in their studies. Moreover, the study revealed that the differences in grade may lead to varying effects on higher-order learning behaviours.

The classrooms of senior grades and clinical teaching more incorporate multiple teaching methods such as problem-based learning (PBL), case teaching and role play. These methods encourage students to complete learning tasks independently and lead to more frequent use of higher-order learning behaviours. As a result, the quality and frequency of senior students’ higher-order learning behaviours are greatly improved compared to those in lower grades. The studies on the higher-order learning in China’s undergraduate education pointed out that, the undergraduate curriculum for junior grade students emphasized more on lower-order thinking, while curriculum for senior grade students emphasized more on higher-order thinking.

### 4.2. The status of learning strategies

With regard to learning strategies, the study revealed that the enquiry-based learning strategy score of medical students in Chinese provincial undergraduate colleges exceeded the norm level. The grade was found to be a significant factor influencing the learning strategies. Specifically, the application of learning strategies changed as the grade progressed. Freshmen and junior students generally exhibited weaker higher-order learning abilities, particularly in the skills necessary for clinical courses, such as integrating information to generate new ideas, evaluating perspectives or conclusions, and applying theoretical concepts to practical problems or novel settings. However, over time and with more advanced learning, the students’ clinical knowledge and skills continued to improve. As they progressed into senior grades, students were exposed to more professional and practical courses that emphasized integration, enquiry-based learning and collaboration, thereby stimulating and encouraging thoughtful learning [10].

The gender was found to be a significant factor affecting learning strategy preference. Specifically, females tended to score higher in receptive learning, while males scored higher in enquiry-based learning. This suggests that females may have a greater ability to process knowledge and attend to details in classroom learning, while males may be more adept at generating divergent thoughts. These findings support the notion that males and females possess different strengths when it comes to various learning strategies, which has been established in previous research.

### 4.3. The influence of learning strategies on higher-level learning behaviour

The results of the study indicate that among the five learning strategies examined, reflective learning strategies had no significant effect on the development of higher-order learning behaviours. Conversely, the other four learning strategies, namely receptive learning, enquiry-based learning, integrative learning and collaborative learning, were found to exert a positive influence on higher-order learning behaviours, with receptive learning having the greatest impact followed by enquiry-based learning, integrative learning and collaborative learning in that order.

First, the results of the study demonstrate that receptive learning strategy serves as the foundation for the development of higher-order learning behaviours among medical students from provincial undergraduate colleges. The high-quality teaching and teacher–student interaction are the prerequisite for high-order learning. It can be attributed to the influence of traditional Chinese learning habits among these students, Chinese students are accustomed to using receiving learning strategies to obtain professional knowledge. The results of the evaluation of receptive learning strategies, as shown in Table 2, indicate that the strategies have a positive impact on higher-order learning, outperforming the norm and demonstrating their significance as a basic function for undergraduate students in China to achieve higher-order learning states.

Second, the study found that enquiry-based learning strategy served as a transformative factor in the development of higher-order learning behaviours among medical students. The learning process in the medical profession is not merely a matter of intensive training, but it is rather a process of self-development. In order to succeed in this process, students must not only focus on acquiring and processing knowledge and practicing technical skills, but they must also pay attention to their own learning experiences [11]. The students are encouraged to develop self-directed learning through initiative and creativity, make use of their previous learning experiences, and acquire the ability to solve complex clinical situations. These are crucial components in transforming the knowledge obtained through receptive learning into practical skills.

Third, the study found that integrative learning strategy served as an advancing factor in the development of higher-order learning behaviours among medical students. As a defining characteristic of higher-order learning, integrative learning involves the mastery of basic subject knowledge and the subsequent development of comprehensive application skills [12]. Guided by basic knowledge and clinical skills training, integrative learning seeks to build connections between existing knowledge and clinical practice, playing a crucial role in solving complex problems in clinical settings. The development of integrative learning must be centred on higher-order cognitive goals in clinical thinking, such as analysis, comprehensive judgement and application, thus gradually transforming the learning needs, learning thinking patterns and clinical skills training methods [13].

Fourth, the study found that collaborative learning strategy was a necessary condition for the achievement of higher-order learning behaviours among medical students. In addition to individual student efforts, the development of higher-order learning behaviours also requires students to consider the emotional environment in clinical settings. This necessitates the establishment of a new relationship between students and teachers, as well as among students, characterized by a ‘learning partner’ dynamic. This approach can enhance students’ cognitive development and improve the quality and frequency of interaction between students and learning partners [14]. Students practice collaborative learning during clinical practice and engage in extracurricular learning experiences to further enrich and enhance learning outcomes.

The fact that the reflective learning strategy was not included in the regression equation highlights that the learning strategies and behaviours of students are influenced by a variety of factors, including their daily habits and the environment in which they are learning. There are significant differences between Chinese universities and those in Europe and the United States in terms of teaching objectives and topic participation [15]. Compared to American undergraduates, Chinese college students do not have a habit of reflecting on their learning, with most choosing to do so only occasionally, as revealed by the 2010 NESS survey [16]. This lack of motivation to act limits the development of critical learning behaviour and innovation in Chinese students. In many Chinese provincial colleges and universities, teaching goals often only focus on low-level strategies like ‘memory and understanding,’ which hinders the students’ ability to think critically and innovate. Medical school courses are particularly affected, with a lack of deep learning environments and challenging tasks that promote reflective learning strategies. As a result, students lack the necessary skills to use reflective learning methods to improve their learning experience [17].

The findings of this study underscore the importance of four influential learning strategies in developing higher-order learning behaviours. To make the most of limited resources, medical education must establish a comprehensive teaching concept that integrates various resources to help students improve both their academic and social abilities through basic knowledge and clinical training [18]. Students must be supported in their transformation towards higher-order learning by integrating professional knowledge, application skills, interpersonal communication, emotional management and other elements into the curriculum system [19]. Both in the preclinical learning stage and the clinical training stage, medical schools must provide support for students from cultural, psychological and learning adaptation perspectives, to facilitate the understanding and application of higher-order learning strategies. Additionally, students must be encouraged to actively participate in the process of creating independent cognitive processing and building knowledge frameworks, stimulating their desire and potential for self-directed learning and optimizing their learning modes as soon as possible [20].

### 4.4. Research limitations and improvement directions

The limitations of this study can be displayed as follows: first, the conclusion regarding the impact of receptive learning, enquiry-based learning, integrative learning and collaborative learning strategies on higher-order learning behaviours among medical students in provincial undergraduate colleges in China was primarily based on subjective evaluations by the students themselves. Therefore, it is essential to improve the objectivity of these conclusions through further verification [21]. Second, due to the cross-sectional design of this study, the preference for using different learning strategies among students may change as they progress through their grade or accumulate more learning experiences. It is unclear why such changes on the formation of higher-order learning behaviours have an impact at this time.

Additionally, future research could employ quantitative methods such as experimental or quasi-experimental design to verify the impact of different learning strategies on higher-order learning behaviours with more objective evidence. Furthermore, it is also important to consider the influence of various confounding variables, such as student characteristics, teaching methods and institutional culture, on the formation of higher-order learning behaviours. By incorporating these variables into the study, we can gain a more comprehensive understanding of the relationship between learning strategies and higher-order learning behaviours.

## 5. Conclusions

The findings of this study indicate that there are distinct variations in the utilization of learning strategies among students in provincial medical colleges in China. The results revealed that the utilization of inquiry-based, receptive, integrative and collaborative learning strategies has a significant impact on the emergence of higher-order learning behaviours.

Furthermore, the study revealed that the changes in grade influence the frequency and quality of higher-order learning behaviours, as well as the preferences towards reflective and collaborative learning strategies. There are gender differences in the application preferences of receptive and enquiry-based learning strategies among medical students in provincial colleges in China.

These insights contribute to the comprehension of the relationship between learning strategies and higher-order learning behaviours in this population.

## Supplementary Material

Supplemental Material

## Data Availability

Additional data are available upon reasonable request.
