# Supplementary material for: What learning strategies influence higher-order learning behaviours of medical students?
Source: Ann Med. 2023 May 12;55(1):2205166. doi: 10.1080/07853890.2023.2205166 (PMC10184605; doi:10.1080/07853890.2023.2205166)
Supplement: Supplemental Material [file IANN_A_2205166_SM4814.docx]

**Part A: University experience**

| **Serial Number** | **Sequence Number** | **Variable Number** | **Meaning of Variables** | **Variable Assignments*** |
| --- | --- | --- | --- | --- |
| **1. How often did you do the following activities this academic year?** | 1 | A1a | a. You ask questions or participate in discussions in class actively. | 1= Very often  2= Often  3= At times  4= Never |
|  | 2 | A1b | b. You actively answer/think about questions in the class where the teacher has no established answers. |  |
|  | 3 | A1c | c. You are well prepared to give a lecture on a research topic in class. |  |
|  | 4 | A1d | d. You collaborate with other students on assignments or related tasks. |  |
|  | 5 | A1e | e. You will ask other students for help about the course content. |  |
|  | 6 | A1f | f. You help other students understand the course content. |  |
|  | 7 | A1g | g. You did not complete the required assignments or related reading before class. |  |
|  | 8 | A1h | h. You will question the teacher’s point of view in the class. |  |
|  | 9 | A1i | i. You take notes on the key points in class. |  |
|  | 10 | A1j | j. You concentrate on listening to the teacher’s explanation in class. |  |
|  | 11 | A1k | k. You discuss the course content with your classmates after class. |  |
|  | 12 | A1l | l. You use online media to discuss or complete assignments (such as online classes, online forums, chat tools, etc.). |  |
|  | 13 | A1m | m. You can get timely feedback from teachers on your exams and assignments. |  |
| **2. This academic year, has your course emphasized the following areas?** | 14 | A2a | a. Recite the course content. | 1= Highly  2= Normal  3= Partly  4= De-emphasis |
|  | 15 | A2b | b. Apply concepts, theories or methods to practical problems or new situations. |  |
|  | 16 | A2c | c. Understand what constitutes an opinion, experience, or line of reasoning in order to analyze it in depth. |  |
|  | 17 | A2d | d. Emphasis on evaluating an opinion, conclusion, or source of information. |  |


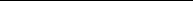


*If there is no special note, in items with code assignment ≤7, 8 uniformly represents multiple choice, 9 uniformly represents overflow; in items with code assignment>7, 98 uniformly represents multiple choice, 99 uniformly represents overflow.

|  | 18 A2e | e. Emphasis on collecting different information to form new perspectives or understandings. |  |
| --- | --- | --- | --- |
| **3. How difficult/challenging was the main course of your major this academic year for you?** | 19 A3 | How difficult has it been for you this academic year in your specialized courses? | 1= Easy  2= Difficult |
| **4. How often did you do the following activities this academic year?** | 20 A4a | a. You go to the library/study room. | 1= Very often  2= Often  3= At times  4= Never |
|  | 21 A4b | b. You take part in lectures or academic reports of interest |  |
|  | 22 A4c | c. You discuss course-related content with teachers after the class. |  |
|  | 23 A4d | d. You need to work very hard to meet the course requirements. |  |
|  | 24 A4e | e. You discuss the number of assignments or grades with your teacher. |  |
|  | 25 A4f | f. You can integrate content from different courses in your assignments. |  |
|  | 26 A4g | g. You can quickly identify key information from extensive reading. |  |
|  | 27 A4h | h. You review your notes after class. |  |
|  | 28 A4i | i. You summarize what you have learned in the course. |  |
|  | 29 A4j | j. You can independently analyze numerical information (such as figures, graphs, data, etc.) and draw conclusions. |  |
|  | 30 A4k | k. You can use digital information to analyze realistic problems (such as price changes, environmental problems, etc.). |  |
|  | 31 A4l | l. You can evaluate and judge the numerical information and conclusions given by others. |  |
| **5. In general, how much books did you read this academic year?** | 32 A5a | a. Textbook or reference book | 1= 0  2= 1-4  3=5-10  4= 11-20  5= More than 20 |
|  | 33 A5b | b. Academic papers/research reports |  |
|  | 34 A5c | c. Non-teaching books (to broaden knowledge) |  |
| **6. How many papers, reports or other curricular writing assignments of the following length have you submitted this academic year?** | 35 A6a | a. Long curricular paper/report (article) (more than 5000 words) | 1= 0  2= 1-4  3=5-10  4= 11-20 |
|  | 36 A6b | b. Medium curricular paper/report (article) (2000- 5000 words) |  |
|  | 37 A6c | c. Short curricular paper/report (article) (less than 2000 words) |  |

| **(Including unfinished ones)?** |  |  | 5= More than 20 |
| --- | --- | --- | --- |
| **7. This academic year, have your teachers done as follows?** | 38 A7a | a. He or she clearly explains the course objectives. | 1= Completely  2= Basically  3= A little  4= No |
|  | 39 A7b | b. He or she precisely states the course requirements. |  |
|  | 40 A7c | c. He or she can reasonably arrange the teaching content |  |
|  | 41 A7d | d. He or she uses examples or diagrams to explain difficulties. |  |
|  | 42 A7e | e. He or she helps with assignments. |  |
|  | 43 A7f | f. He or she helps with learning methods and thinking methods. |  |
|  | 44 A7g | g. He or she gives timely feedback on exams and assignments. |  |
|  | 45 A7h | h. He or she can stimulate your interest in learning. |  |
|  | 46 A7i | i. He or she stimulates your learning initiative. |  |
|  | 47 A7j | j. He or she gives students a degree of autonomy in the teaching process. |  |
|  | 48 A7k | k. He or she encourages students to ask questions. |  |
|  | 49 A7l | l. He or she inspires students to think. |  |
|  | 50 A7m | m. He or she emphasizes problem analysis and resolution. |  |
| **8. How was your communication with the teachers this academic year?** | 51 A8a | a. You discuss your career plans and ideas with your teacher. | 1= Very often  2= Often  3= At times  4= Never |
|  | 52 A8b | b. You discuss your career plans and ideas with your counselor/teacher. |  |
|  | 53 A8c | c. You and your teacher discuss issues such as outlook on life and values. |  |
|  | 54 A8d | d. You discuss issues such as outlook on life and values with the counselor/teacher. |  |
|  | 55 A8e | e. You and your teacher are involved in work outside the course (such as club activities, student union, etc.). |  |
| **9. This academic year, is your course paper/report required to ...?** | 56 A9a | a. Present your own point of view or idea and make an argument | 1= Very often  2= Often  3= At times  4= Never |
|  | 57 A9b | b. Discuss with teacher/classmates repeatedly |  |
|  | 58 A9c | c. Extensive collect and review of information |  |
|  | 59 A9d | d. Cite of many relevant literature and data in-depth |  |
| **10a. This academic year,** | 60 A10aa | a. Closed-book exam | 1= Very often |

| **what is the usual frequency of assessment methods for your main courses?** | 61 A10ab | b. Open book or semi-open book exam | 2= Often  3= At times  4= Never |
| --- | --- | --- | --- |
|  | 62 A10ac | c. Course Papers/Reports/Works/Designs/Proposals |  |
|  | 63 A10ad | d. Operations and Demonstrations in class |  |
|  | 64 A10ae | e. Assignments |  |
|  | 65 A10af | f. In-class quiz |  |

**10b. Among the options above, what was the assessment method for the course that helped you the most? (Choose two only)**

66 A10b Among the options above, what was the most helpful way of assessing your learning outcomes? (Choose two only)

1=a

2=b

3=c

4=d

5=e

6=f

| **11. Do you agree with the following statement about your main course assessment?** | 67 A11a | a. I don’t need to study all the required content of the course, just study the key points to get a high score. | 1= Totally agree  2= Agree  3= Disagree  4= Totally disagree |
| --- | --- | --- | --- |
|  | 68 A11b | b. In the process of completing the assignment, even if there is no total understanding, I can still get a high score. |  |
|  | 69 A11c | c. I can get enough teacher feedback to tell me what to do. |  |
|  | 70 A11d | d. I can get feedback from the teacher that I know why I got this grade. |  |
|  | 71 A11e | e. Through teacher feedback I know how to improve next learning. |  |
|  | 72 A11f | f. After the exam, most of the content may be forgotten quickly. |  |
| **12. How often did you do the following activities this academic year?** | 73 A12a | a. I visit exhibitions and watch performances such as plays, dances, etc. | 1= Very often  2= Often  3= At times  4= Never |
|  | 74 A12b | b. I reflect on and evaluate my own learning process. |  |
|  | 75 A12c | c. Through learning, I have changed my understanding of a problem/concept. |  |
|  | 76 A12d | d. I challenge my pre-existing view of some problems. |  |
|  | 77 A12e | e. I understand other people’s perspectives better through transpositional consideration. |  |
|  | 78 A12f | f. I am able to consider problems from different perspectives in class discussions or assignments. |  |
|  | 79 A12g | g. I reflect/examine the strengths and weaknesses of my perspective. |  |

|  | 80 A12h | h. I relate my learning to social issues. |  |
| --- | --- | --- | --- |
|  | 81 A12i | i. I relate ideas from the course to my own experience and knowledge. |  |
|  | 82 A12j | j. I learn the course online. |  |
| **13. How often have you communicated with the following groups this academic year?** | 83 A13a | People of different races or nationalities | 1= Very often  2= Often  3= At times  4= Never |
|  | 84 A13b | People from different economic backgrounds |  |
|  | 85 A13c | People of different religions |  |
|  | 86 A13d | People from different geographical origin |  |
|  | 87 A13e | People from different countries |  |
| **14. How are your interpersonal relationship in college?** | 88 A14a | a. Your relationship with classmates | 1= unfriendly/ no mutual support  7= friendly/mutual support |
|  | 89 A14b | b. Your relationship with teachers | 1=I can’t find them/no help when needed.  7=I can find them/help when needed. |
|  | 90 A14c | c. Your relationships with student system staff (e.g., counselors) | 1=I can’t find them when needed/they doesn’t understand me.  7 = I can find them when needed/they understand me. |
|  | 91 A14d | d. Your relations with administrative staff (such as the office of academic affairs, etc.). | 1=I can’t find them when needed/they doesn't understand me.  7 = I can find them when needed/they understand me. |
| **15. Which of the following activities have you done, or plan to do before graduation?** | 92 A15a | a. To take part in internship, social practice or survey. | 1= Have done  2= Intend to do  3= Do not intend to do  4= Have not decided yet |
|  | 93 A15b | b. Joining community service or volunteering. |  |
|  | 94 A15c | c. Submission to professional academic journals/academic conferences, etc. |  |
|  | 95 A15d | d. To participate in study clubs (such as reading clubs, English clubs, etc.). |  |
|  | 96 A15e | e. To do research with teachers. |  |
|  | 97 A15f | f. To learn a language beyond curriculum requirements (such as minor in second foreign language in New Oriental Education & Technology Group, etc.). |  |

|  | 98 A15g g. To study abroad (Short or Long Term). |  |
| --- | --- | --- |
|  | 99 A15h h. To participate in various academic, professional, entrepreneurial or design competitions. |  |
|  | 100 A15i i. To apply for professional qualification certificate/skill level certificate. |  |
|  | 101 A15j j. To apply for second degree/major minor |  |
| **16. Does your university emphasize the following?** | 102 A16a a. Students are expected to devote significant time to their studies. | 1= Highly  2= Normal  3= Partly  4= De-emphasis |
|  | 103 A16b b. The college provides support and assistance for your studies (such as academic guidance, writing centers). |  |
|  | 104 A16c c. The college provides support and services for your physical and mental health (such as medical care, psychological counseling, etc.). |  |
|  | 105 A16d d. The college provides guidance and assistance for your job hunting. |  |
|  | 106 A16e e. The college encourages students from different areas, ethnic groups and family backgrounds to contact each other. |  |
|  | 107 A16f f. The college provides social contact opportunities. |  |
|  | 1108 A16g g. Students' participation in various campus cultural and sports activities (such as art performances, sports competitions, etc.). |  |
|  | 109 A16h h. The college helps you cope with financial problems and complete your studies. |  |
|  | 110 A16i i. The participation in activities related to major social, economic and political issues. |  |
|  | 111 A16j j. The application of computer during study. |  |
|  | 112 A16k k. Online learning/Distance learning |  |
| **17. Do you agree with the following description of your studies?** | 113 A17a a. Learning is to increase the amount of knowledge through memory. | 1= Totally agree  2= Agree  3= Disagree  4= Totally disagree |
|  | 114 A17b b. To learn is to help us know and understand the world. |  |
|  | 115 A17c c. Learning is a process of discovery and inquiry. |  |
|  | 116 A17d d. Learning is the process of vocational training. |  |
|  | 117 A17e e. Learning is the process of self-development and growth as a true human being. |  |
|  | 118 A17f f. When I encounter difficulties in learning, I will always try my best to overcome them. |  |
|  | 119 A17g g. I am filled with joy when I focus on my studies. |  |
|  | 120 A17h h. I am willing to learn because it keeps me growing. |  |
|  | 121 A17i I. Most of the time, I don't know what the things I have learned mean to me. |  |
| **18. How motivated have you been this year?** | 122 A18 How motivated have you been this year? | 1= Very strong |

|  |  |  | 2= Strong  3= Weak  4= Very weak |
| --- | --- | --- | --- |
| **19. What has been your main motivation this academic year?** | 123 A19a | a. I’m interested in what I'm learning. |  |
|  | 124 A19b | b. I want to challenge/improve myself. |  |
|  | 125 A19c | c. Job hunting or further education |  |
|  | 126 A19d | d. To meet teacher and parent expectations. |  |
| **20. How many hours of sleep did you get on average this academic year?** | 127 A20 | How many sleeping hours did you get on average this academic year? | 1= 3  2= 3-4  3= 4-5  4= 5-6  5= 6-7  6= 7-8  7= More than 8 |
| **21. In general, how much time do you spend on the following activities a week (7 days) this academic year?** | 128 A21a | a. Attendance (total hours per week) | 1= 0  2= 1-5  3=6-10  4=11-15  5= 16-20  6= 21-25  7= 26-30  8= More than 30 |
|  | 129 A21b | b. Study time outside of class (e.g., preview, review, doing homework/labs, reading literature, etc.) (total hours per week) |  |
|  | 130 A21c | c. Part-time job (on-campus/off-campus) (total hours per week) |  |
|  | 131 A21d | d. Participate in extracurricular activities (such as student union/youth league committee, club activities, campus publications, etc.) (total hours per week) |  |
|  | 132 A21e | e. Entertainment and socializing (being with friends, playing games, watching TV or videos, chatting online, etc.) (total hours per week) |  |
|  | 133 A21f | f. Fitness, exercise, etc. (total hours per week) |  |
| **22. How often did you do the following activities this academic year?** | 134 A22a | a. To discuss or debate important social, political or philosophical issues. | 1= Very often  2= Often  3= At times |
|  | 135 A22b | b. To present a keynote address in front of a group of people. |  |
|  | 136 A22c | c. Use innovative ideas or methods to solve problems. |  |

|  | 137 A22d | d. To learn about an issue through multiple sources of information (books, magazines, Internet, databases, etc.). |  | 4= Never |
| --- | --- | --- | --- | --- |
|  | 138 A22e | e. Read foreign language articles or books. |  |  |
|  | 139 A22f | f. Listen/watch foreign language audio and video. |  |  |
|  | 140 A22g | g. Communicate in a foreign language (oral or written). |  |  |
|  | 141 A22h | h. Objectively evaluate the solutions to some problems. |  |  |
|  | 142 A22i | i. I discuss complex problems with others and come up with better solutions. |  |  |
|  | 143 A22j | j. Discuss the possible ethical and moral consequences of certain actions. |  |  |
| **23. Has your university life enhanced your development in the following areas?** | 144 A23a | a. Extensive coverage of various fields of knowledge |  | 1= Greatly enhanced  2= Considerably enhanced  3= Only a little enhanced  4= No enhanced |
|  | 145 A23b | b. Rich professional knowledge and skills |  |  |
|  | 146 A23c | c. Good oral presentation skills |  |  |
|  | 147 A23d | d. Good written communication skills |  |  |
|  | 148 A23e | e. Organizational leadership |  |  |
|  | 149 A23f | f. Proficiency in the use of information technology |  |  |
|  | 150 A23g | g. critical thinking |  |  |
|  | 151 A23h | h. Collaborate effectively with others |  |  |
|  | 152 A23i | i. Solve complex problems in reality |  |  |
|  | 153 A23j | j． Self-learning |  |  |
|  | 154 A23k | k. Analytical skills for numerical and statistical  information. |  |  |
|  | 155 A23l | l． Know yourself |  |  |
|  | 156 A23m | m． Establish and clarify personal outlook on life and values. |  |  |
|  | 157 A23n | n． Determine your future development plan. |  |  |
|  | 158 A23o | o. Understand the cultures and values of different groups. |  |  |
| **24. Does the following description fit your situation?** | 159 A24a | a. Seriously, I've never hated anyone. |  | 1= Totally agree  2= Relatively agree |
|  | 160 A24b | b. I have never been jealous of my classmates who are better than me. |  |  |

|  | 161 A24c | c. I’ve never made excuses/put the blame on others for my fault. | 3= Not quite agree  4= disagree |
| --- | --- | --- | --- |
|  | 162 A24d | d . I never get bored when someone says something completely different from mine. |  |
|  | 163 A24e | e. I almost never feel the urge to scold others. |  |
|  | 164 A24f | f. I never hesitate to let go of my own business to help those in need. |  |
| **25. Please fill in your major** | 165 A25a | Major |  |
|  | 166 A25b | Elective/Minor Major |  |
|  | 167 A25c | If you do not have a major yet, please fill in your intended major. |  |
| **26. Are you interested in your major?** | 168 A26 | 26. Are you interested in your major? | 1= Very interested  2= Relatively  3= Only a little  4= no interest |
| **27. Do you think your major will help you lead a satisfying life in the future?** | 169 A27 | 27. Do you think your major will help you lead a satisfying life in the future? | 1= Very helpful  2= Relatively  3= Only a little  4= No help |
| **28a**. **How many credits have you taken this academic year?** | 170 A28a | 28a． How many credits have you taken this academic year? |  |
| **28b. What was your GPA last semester?** | 171 A28b1 | Credit Score (4.0 is the full score) |  |
|  | 172 A28b2 | GPA (100 is the full score) |  |
| **28c**． **Compared with your classmates in the same class/major, your grades last semester is:** | 173 A28c | 28c． Compared with your classmates in the same class/major, your grades last semester is: | 1= Top 5%  2= Top 5%-20%  3=Top 20%-50%  4=50%-80％  5 = Bottom 20% |
| **28d. During college, did you fail any subjects?** | 174 A28d | 28d. During college, did you fail any subjects? | 1= No  2= Yes |

|  | 175 A28db | How many subjects have you ever failed? |  |
| --- | --- | --- | --- |
| **29. Have you joined in any of the following student institutions or organizations? If yes, please fill in the title of the highest position.** | 176 A29a | Youth league committee/Student union | 1= Yes  2= No |
|  | 177 A29ab | The title of the highest position |  |
|  | 178 A29b | College youth league committee/College student union | 1= Yes  2= No |
|  | 179 A29bb | The title of the highest position |  |
|  | 180 A29c | League branch secretary/Class committee | 1= Yes  2= No |
|  | 181 A29cb | The title of the highest position |  |
|  | 182 A29d | Club organization | 1= Yes  2= No |
|  | 183 A29db | The title of the highest position |  |
| **30a． During college, have you obtained the following certificates?** | 184 A30a | During college, have you obtained the following certificates? |  |
|  | 185 A30a1 | College English Test Band 4 (CET-4) | 1= Yes  0= No |
|  | 186 A30a2 | College English Test Band 6 (CET-6) |  |
|  | 187 A30a3 | National Computer Rank Examination (NCRE) |  |
|  | 188 A30a4 | Professional qualification certificate (such as Certified Public Accountant, Legal professional qualification certificate, etc.) |  |
|  | 189 A30a5 | Skill Level Certificate |  |
|  | 190 A30a6 | Others (please fill in the blank) |  |
|  | 191 A30a7 | None of above |  |
|  | 192 A30ab | Please fill in the blank |  |
| **30b． During college, have you received any of the following awards?** | 193 A30b | During college, have you received any of the following awards? |  |
|  | 194 A30b1 | School-level general awards | 1= Yes  0= No |
|  | 195 A30b2 | The highest scholarship at the school-level |  |

|  | 196 A30b3 | Provincial/City awards |  |
| --- | --- | --- | --- |
|  | 197 A30b4 | National awards |  |
|  | 198 A30b5 | International awards (such as various competitions) |  |
|  | 199 A30b6 | Others (please fill in the blank) |  |
|  | 200 A30b7 | None of above |  |
|  | 201 A30bb | Please fill in the blank |  |
| **31. What are you going to do after graduation? (Single choice only)** | 202 A31 | What are you going to do after graduation? | 1= Find a job in China  2= Find a job abroad  3= Study for a doctorate in China  4= Study abroad for a doctorate  5= Study for a master’s degree in China  6= Study abroad for a masterss degree  7= No clear plan |
| **32. Are you satisfied with the following aspects of the university?** | 203 A32a | a. Overall study experience | 1= Very dissatisfied  7= Very satisfied |
|  | 204 A32b | b. Academic experience |  |
|  | 205 A32c | c. Social experience |  |
|  | 206 A32d | d. Learning hardware conditions (classroom, library, laboratory, network, etc.) |  |
|  | 207 A32e | e. Style of study and study atmosphere |  |
|  | 208 A32f | f. Quality of the courses |  |
|  | 209 A32g | g. Teaching level |  |
|  | 210 A32h | h. A second chance to choose a major |  |
|  | 211 A32i | i. Guidance on job hunting |  |
|  | 212 A32j | j. Your overall harvest and growth |  |
| **33. Would you recommend this university to others?** | 213 A33 | Would you recommend this university to others? | 1= No  7= Yes |
